# Toward ‘Computational-Rationality’ Approaches to Arbitrating Models of Cognition: A Case Study Using Perceptual Metacognition

**DOI:** 10.1162/opmi_a_00100

**Published:** 2023-09-20

**Authors:** Yingqi Rong, Megan A. K. Peters

**Affiliations:** Department of Mathematics, University of California, Irvine, Irvine, CA, USA; Department of Cognitive Sciences, University of California, Irvine, Irvine, CA, USA; Center for the Neurobiology of Learning and Memory, University of California, Irvine, Irvine, CA, USA; Program in Brain, Mind, & Consciousness, Canadian Institute for Advanced Research, Toronto, Canada

**Keywords:** perceptual metacognition, computational rationality, computational models, model comparisons

## Abstract

Perceptual confidence results from a metacognitive process which evaluates how likely our percepts are to be correct. Many competing models of perceptual metacognition enjoy strong empirical support. Arbitrating these models traditionally proceeds via researchers conducting experiments and then fitting several models to the data collected. However, such a process often includes conditions or paradigms that may not best arbitrate competing models: Many models make similar predictions under typical experimental conditions. Consequently, many experiments are needed, collectively (sub-optimally) sampling the space of conditions to compare models. Here, instead, we introduce a variant of optimal experimental design which we call a *computational-rationality* approach to generative models of cognition, using perceptual metacognition as a case study. Instead of designing experiments and post-hoc specifying models, we *began* with comprehensive model comparison among four competing generative models for perceptual metacognition, drawn from literature. By simulating a simple experiment under each model, we identified conditions where these models made *maximally diverging predictions* for confidence. We then presented these conditions to human observers, and compared the models’ capacity to predict choices and confidence. Results revealed two surprising findings: (1) two models previously reported to differently predict confidence to different degrees, with one predicting better than the other, appeared to predict confidence in a direction *opposite* to previous findings; and (2) two other models previously reported to equivalently predict confidence showed stark differences in the conditions tested here. Although preliminary with regards to which model is actually ‘correct’ for perceptual metacognition, our findings reveal the promise of this *computational-rationality* approach to maximizing experimental utility in model arbitration while minimizing the number of experiments necessary to reveal the winning model, both for perceptual metacognition and in other domains.

## INTRODUCTION

Perceptual metacognition is the process by which we evaluate our own perceptual capacities: Having decided on the most likely source or identity of an incoming set of sensory signals, we can then evaluate whether that decision is likely to be correct (Fleming et al., 2012; Pouget et al., 2016). Many generative computational models have been put forth to describe how this metacognitive evaluation may occur, ranging from Bayesian ideal observers (Adler & Ma, 2018a, 2018b; Peters & Lau, 2015) to heuristic models (Maniscalco et al., 2016, 2021), process models (Braun et al., 2018; Kiani et al., 2014; Pleskac & Busemeyer, 2010; Zylberberg et al., 2012, 2016), and many others. Indeed, many of these models (including those which actually differ on fundamental levels) appear to enjoy great empirical support—especially from studies designed to test precisely the authors’ favorite model against one or two alternatives.

Yet herein lies an insidious problem. Typically, studies of perception, cognition, metacognition, and so on may be designed such that a behavioral experiment is conducted, and then several models are fit to the resulting data. Sometimes, the authors’ target model ‘wins’ a model comparison against a null model or a few previously-published competing frameworks (Adler & Ma, 2018a; Maniscalco et al., 2016; Peters, Fesi, et al., 2017; Peters, Thesen, et al., 2017; Shekhar & Rahnev, 2021). Other times, however, a model comparison might result in equivocal results, with two or more models potentially explaining results approximately equally well (Aitchison et al., 2015; Peters & Lau, 2015). Arguably, in such cases the behavioral paradigm was not optimized to specifically arbitrate between competing viewpoints, and instead represented a pseudorandom sample of possible approaches to revealing the computational mechanisms of perception or metacognition.

What should we as scientists do instead? *Computational rationality* (Gershman et al., 2015) suggests that any intelligent organism must consider the expected value of an action with reference to the cost of a computation. The idea has been introduced as a framework for describing bounded utility maximization (Lewis et al., 2014), and as a linking framework spanning the fields of artificial intelligence, neuroscience, and cognitive science (Gershman et al., 2015). Indeed, utility maximization drives behavior across many diverse domains, from foraging (Kamil, 1983; Pyke, 1984) to attention-driven saccadic eye movements (Gottlieb & Oudeyer, 2018; Gottlieb et al., 2013). That is, intelligent organisms appear to aim to maximize the utility of their actions and minimize the cost, under biological and temporal constraints. As intelligent organisms, we scientists ought to do the same thing when it comes to arbitrating models of cognitive processes: We should design experiments to *maximally* arbitrate competing frameworks while minimizing the cost (effort, funding) necessary to do so as a variant of optimal experimental design (Kiefer, 1959; Smucker et al., 2018). Here, we use perceptual metacognition as a case study to explore the potential power of such an approach.

In optimal experimental design, a numerical criterion is chosen to select the conditions, number of trials, factor combinations, and so on to minimize either the variance of factor effects or the average prediction variance across the design region (Smucker et al., 2018). Similar approaches have been taken recently to identify stimuli that can best differentiate between deep learning network architectures as the best models of humans perceptual judgments (dubbed “controversial stimuli”; Golan et al., 2020). Relatedly, so-called “active experimental design” (Blanchard & Sapsis, 2021) seeks to drive active learning algorithms towards inputs which would be most informative for self-driven learning.

Here, we extend these ideas to include competing generative modeling frameworks coupled with (human) experimenter control in the study of perceptual decisions and metacognition. In particular, instead of performing a series of experiments and then specifying and fitting several computational models, we should specifically design our experiments to *maximize utility* (i.e., have the best chance of arbitrating those models) while *minimizing the cost* (i.e., minimizing the number of experiments or conditions necessary). In other words, we should aim to design experiments that target the exact processes, behavioral effects, or neural correlates where two or more models are fundamentally incompatible. This goal is a core tenet of meta-scientific approaches such as “adversarial collaborations” (Cohen et al., 2000; Cowan et al., 2020), but we should also aim to employ such practices even in our own individual research groups. In the study of perceptual metacognition, this would amount to exploring multiple candidate models (either analytically or through simulation) *before* conducting any experiments, and then specifically designing such experiments to present conditions or paradigms where two or more models make diverging predictions. These are the conditions which have the greatest expected utility, and from which we should select a minimal target number to actually conduct in order to minimize the costs (i.e., minimize the number of experiments necessary to reveal the ‘best’ model).

Here we took as first case study of this approach four competing models of perceptual metacognition: (1) marginalizing “ideal observer” Bayesian models described by Peters and Lau (2015), (2) the “hierarchical ideal observer” described by Peters and Lau (2015), (3) a second “ideal observer” model described by Aitchison and colleagues (2015), and (4) the “response-congruent evidence” model described by several groups (Maniscalco et al., 2016; Peters, Thesen, et al., 2017, also called the ‘max’ model; Aitchison et al., 2015). Rather than conducting an experiment covering multiple possible conditions and then fitting all four models post-hoc, we *began* by simulating all models and selecting the conditions at which two or more models maximally diverged in their predictions for confidence judgments; we refer to these conditions as the *utility-maximizing conditions*. We then conducted an experiment presenting stimuli from these conditions to human observers in a perceptual decision-making + confidence judgment task, and compared model fits. We found that our approach revealed differences between two models previously thought to equivalently explain perceptual metacognition behavior (both from Peters and Lau (2015)), and if anything, two other models (from Aitchison et al., 2015) showed trends in the opposite direction from previously reported in that investigation. Our findings suggest that the *computational-rationality* approach to model arbitration is a powerful framework for revealing novel insight into the computations underlying perceptual metacognition, further suggesting that future work in other domains could similarly significantly benefit from adopting a utility-maximization approach to scientific model arbitration.

## METHODS

### Computational Models

The task to be performed by the human observers is a 3-alternative forced choice in which three stimuli are shown simultaneously. These stimuli could be anything in principle, but here we assume that the stimuli are perceptual stimuli with varying ‘strengths’ (e.g., contrast, motion coherence; Britten et al., 1992). (See Procedure section for details about the stimuli used in this experiment.) The observer must judge which of the three stimuli is ‘strongest’ and then rate confidence.

The true strength (e.g., coherence for a random-dot kinematogram) of the combination of the three stimuli, *c*, is a hidden variable from the perspective of the observer. That is, there is a generating distribution for the ‘source’ *S* representing all three stimulus strengths simultanously which is a trivariate normal distribution centered at the true stimulus strengths, *c* (a point in 3-dimensional space):

$$S∼N\left(c,\Sigma \right)$$ (1)

with covariance matrix Σ the 3 × 3 identity matrix (for generalizability to cases where covariances are nonzero, in keeping with recent work; Miyoshi & Lau, 2020; Webb et al., 2023) and

$$c=\left[c_{1},c_{2},c_{3}\right]$$ (2)

such that each dimension of *c* represents the true strength of each of the stimuli presented simultaneously on the screen.

The location *c* of the generating stimulus thus indicates which of the three stimuli is strongest; in a perfect (noiseless) world, this would amount to

$$i=\underset{i}{\text{argmax}}\left(c\right)$$ (3)

that is, the largest element of *c* indicates which of the three stimuli possesses strongest evidence. An ideal case of this, for example, could be a ‘source’ *S* (a random variable centered at *c*) for which one stimulus—the *target*—contains signal (*c*_*target*_ > 0) and the other two (*distractors*) do not (*c*_*distractors*_) = 0). Therefore, assume the observer uses these ‘canonical stimuli’ as points of comparison for the stimulus at hand to select which idealized ‘source’ *S* (with one target and two 0-strength distractors) might best explain the observed data:

$$\begin{array}{l} S_{1}∼N\left(\left[c_{1},0,0\right],\Sigma \right)\\ S_{2}∼N\left(\left[0,c_{2},0\right],\Sigma \right)\\ S_{3}∼N\left(\left[0,0,c_{3}\right],\Sigma \right) \end{array}$$ (4)

where again each distribution is a trivariate normal distribution. For the purposes of the Bayesian computations below (in all models), we assume equal prior probability for all three stimuli to become the target, that is, *p*(*S* = *S*_1_) = *p*(*S* = *S*_2_) = *p*(*S* = *S*_3_) = 1/3 (this assumption could be relaxed, however, if for example one were to design a task such that one stimulus location were more likely to contain a target than the other two). These can then represent the ‘best case scenario’ ideal cases that an observer might wish to weigh evidence against, according to the process described for each model individually, below.

Finally, because *c* is hidden and the observer has internal noise, the observer only has access to a noisy sample *d* that is generated by the (3-patch) stimulus centered at *c*, that is,

$$d∼N\left(c,\Sigma \right)$$ (5)

These foundations are shared by all four models tested. The models differ in their definitions of how type 1 decisions and type 2 confidence judgments are made. However, it is important to note that even though the algorithm (i.e., *equations*) by which the type 1 decision is made may differ across the four models, they all nevertheless should make the same type 1 choices; this is because the type 1 decision under the constraints presented here—equal prior probability, equal variance, and zero covariance—collapses onto essentially deciding which element of *d* is largest, or inferring which element of *c* was largest (given that the observer does not have access to *c*, only *d*). (Do note that if the *a priori* probabilities for each ‘canonical’ source *S* were *not* equal (i.e., *p*(*S* = *S*_1_) ≠ *p*(*S* = *S*_2_) ≠ *p*(*S* = *S*_3_)), the task would not collapse in such fashion.) For completeness, however, we present each model’s definitions of type 1 behavior below. We then show how each model makes type 2 judgments differently.

#### Model 1: Marginalizing Bayesian Ideal Observer (“P&L-IO”).

The ideal type 1 decision (here, “P&L-IO”; Peters & Lau, 2015) is that which maximizes the posterior probability of *S* given the data observed. That is, since *c* is unknown for the subject (with the prior over *c*, *p*(*c*) assumed to be flat across the stimulus space), we include all possible marginalized combinations of *c* in the signal space to calculate the joint probability of *S* and *c* to know the stimulus with the highest evidence (e.g., stimulus with highest motion coherence) *S*.

$$\begin{array}{l} p\left(S,c|d\right)=\frac{p\left(d|S,c\right)p\left(S,c\right)}{p\left(d\right)}\\ \;\to p\left(S|d\right)=\int p\left(S,c|d\right)dc \end{array}$$ (6)

Then, the observer makes a type 1 decision about which stimulus (*S*_1_, *S*_2_, or *S*_3_) had the strongest signal based on which posterior estimate is highest.

$$\text{choice}=\underset{i}{\text{argmax}}\left(p\left(S_{i}|d\right)\right),\;i\in 1,2,3$$ (7)

P&L-IO then defines type 2 confidence judgments as a direct readout of the posterior probability used to make the type 1 decision. That is,

$$\text{conf}=p\left(S_{\text{choice}}|d\right)$$ (8)

The type 2 ratings thus represent the expected value of type 1 correctness.

#### Model 2: Hierarchical Bayesian Ideal Observer (“P&L-H”).

The P&L-IO model supposes that the observer does not have access to the exact stimulus strength which produced the observed data, and thus the observer marginalizes over all possible source stimulus strengths *c* to make type 1 and type 2 judgments. An alternative strategy is that the observer makes an inference about the most likely stimulus strength in each of the three stimulus patches to have given rise to observed data, and then uses the results of this inference process to subsequently make type 1 and type 2 judgments.

Thus, instead of marginalizing over all possible values of *c*, Model 2 (“Hierarchical Bayesian ideal observer”; P&L-H; Peters & Lau, 2015) forms the type 1 judgments by inferring the generating distributions to be centered at *c*_*i*_ = *d*_*i*_, that is, each inferred source along each possible axis (stimulus location) is the most likely coherence (evidence) level *c*_*i*_ to have generated stimulus strength *d*_*i*_ at that location. Algorithmically, this amounts to ‘projecting’ each element of *d* onto each of the axes in the 3-dimensional space to find three source generating distributions for which one element is nonzero and the other two elements are zero, that is, [$c_{1}^{*}$, 0, 0], [0, $c_{2}^{*}$, 0], and [0, 0, $c_{3}^{*}$]:

$$c_{i}^{*}=d_{i}$$ (9)

The type 1 decision is then found by computing the posterior probability of each source having been the ‘strongest’ via:

$$p\left(S,c^{*}|d\right)=\frac{p\left(d|S,c^{*}\right)p\left(S,c^{*}\right)}{p\left(d\right)}$$ (10)

Then, as above, the observer makes the type 1 decision based on which posterior estimate is highest.

$$\text{choice}=\underset{i}{\text{argmax}}\left(p\left(S_{i},c_{i}^{*}|d\right)\right),\;i\in 1,2,3$$ (11)

P&L-H then defines type 2 confidence judgments again as a direct readout of the posterior probability used to make the type 1 decision. That is,

$$\text{conf}=p\left(S_{\text{choice}}^{*},c^{*}|d\right)$$ (12)

As above, the type 2 ratings thus represent the expected value of type 1 correctness.

#### Model 3: 3-point Bayesian Ideal Observer (“A-IO”).

While the P&L-IO and P&L-H models suppose that the observer uses a three-dimensional estimate *c* to make both type 1 and type 2 judgments, the 3-point Bayesian ideal observer (A-IO) (Aitchison et al., 2015) model supposes that the observer will only use a one dimensional vector of source stimulus strength. In other words, the observer assumes the source distributions along each possible stimulus dimension—that is, the stimulus present at each possible stimulus location on the screen—must haveequal strength. That is,

$$c_{1}=c_{2}=c_{3}$$ (13)

where we include possible marginalized enumeration of *c* = [*c*_1_, *c*_2_, *c*_3_] in the signal to calculate the joint probability. Remaining logic follows that of the P&L-IO model.

The decision is that which maximizes the posterior probability of *S* given the data observed. That is, since (as above) *c* is unknown for the subject, we include all possible marginalized combinations of *c* in the signal space to calculate the joint probability of *S* and *c* to know the most coherent stimulus *S*.

$$\begin{array}{l} p\left(S,c|d\right)=\frac{p\left(d|S,c\right)p\left(S,c\right)}{p\left(d\right)}\\ \;\to p\left(S|d\right)=\int p\left(S,c|d\right)dc \end{array}$$ (14)

Then, the observer will make a type 1 decision about which patch (*S*_1_, *S*_2_, or *S*_3_) had the strongest signal between based on which posterior estimate is highest.

$$\text{choice}=\underset{i}{\text{argmax}}\left(p\left(S_{i}|d\right)\right),\;i\in 1,2,3$$ (15)

A-IO then defines type 2 confidence judgments as a direct readout of the posterior probability used to make the type 1 decision. That is,

$$\text{conf}=p\left(S_{\text{choice}}|d\right)$$ (16)

The type 2 ratings thus again represent the expected value of type 1 correctness under this scheme.

#### Model 4: Response-Congruent Evidence Model (“RCE”).

Finally, Model 4 (“Response-congruent evidence model”; RCE; Aitchison et al., 2015; Maniscalco et al., 2016) is a heuristic model which takes as confidence the independent magnitude of evidence supporting the choice that was made, regardless of evidence favoring other possible choices. This model has also been called the ‘max’ model by Aitchison and colleagues (2015), and is related to heuristic models discussed by Locke and colleagues (2022).

The RCE model makes type 1 decisions simply by taking which element of *d* is largest:

$$\text{choice}=\underset{i}{\text{argmax}}\left(d_{i}\right)$$ (17)

RCE then rates confidence according to the magnitude of evidence along the *chosen dimension*, that is,

$$\text{conf}=d_{\text{choice}}$$ (18)

This is referred to as the magnitude of ‘response-congruent’ evidence or sometimes of ‘decision-congruent evidence’ in previous explorations of this model (Maniscalco et al., 2016; Peters, Thesen, et al., 2017).

### Model Simulations

We used Monte Carlo simulations to make type 1 and type 2 predictions for each of the four models described above. The range of values the strength of a signal (*c*) was allowed to take was from 0 to 5, with a step size of 0.5, resulting in 11 possible values. (Note that these are arbitrary units, and choice of signal strength could also be scaled up or down if noise (Σ) were similarly scaled.) This involves simulating 11 × 11 × 11 possible ‘source’ points (1331 points total) for the actual stimulus shown, *S*, within a three-dimensional grid, with evidence favoring a particular stimulus forming each axis. At each point we generated samples from a trivariate normal generating distribution (with Σ the 3 × 3 identity matrix as described above) to represent the combination of three stimulus strengths, to be shown as a 3-alternative forced choice in the behavioral experiments. Each point thus formed the mean of a trivariate normal generating distribution as defined above. At each point we simulated 10,000 trials and produced type 1 choices and type 2 confidence ratings for each model tested. Model simulations were performed through custom scripts written in Python version 3.8.2.

### Selecting Utility-Maximizing Conditions

Our primary goal with this study is to identify points in the 3-dimensional stimulus space where models maximally diverge in their type 2 predictions (e.g., points where one model predicts high confidence while another predicts low confidence). Probing participants’ choices and confidence judgments at these points provides us with the best chance of discovering whether one of the four models tested can better capture human performance over the other models. We refer to these as the *utility-maximizing conditions*. After simulating all four models using the definitions above and recording type 1 decisions and type 2 confidence judgments at all simulated points (see Model Simulations section), we then z-scored all confidence judgments within a given model across all simulated points in the 3-D stimulus space such that all confidence judgments across all four models would be in the same scale. This is necessary because while three of the models (the three “ideal observer” type models) produce confidence judgments in the range of 0–1 due to their output as posterior probabilities, the fourth model (RCE) produces confidence judgments in the range of stimulus ‘strengths’ simulated by the model.

Across all these points, we then proceeded with pairwise comparisons between each pair of models. At each simulated point, we computed Cohen’s *d* between pairs of models *i* and *j* (*d* = $\frac{\mu_{j}-\mu_{i}}{\sqrt{\left(\sigma_{i}^{2}+\sigma_{j}^{2}\right)/2}}$) as a measure of effect size for the difference between z-scored confidence predictions for that pair of models at that simulated point in stimulus space. We selected three points in stimulus space which produced maximal summed Cohen’s *d* across all pairs of models (see Utility-Maximizing Conditions section), and used these to drive stimulus creation for the behavioral experiment. We note that in this condition selection process, the simulations were completed with Σ = *I*3, the 3-dimensional identity matrix—that is, all variances were set to 1 and covariances to 0. However, we also confirmed that the choice of variance did not change which conditions would be selected as utility-maximizing by re-running the entire procedure with two additional Σ options: Σ = 0.5 *I3 = [0.5 0 0; 0 0.5 0; 0 0 0.5] and Σ = 1.5 * *I*3 = [1.5 0 0; 0 1.5 0; 0 0 1.5]. In both instances, the same rank-ordering of Cohen’s d was obtained, leading to selection of the same utility-maximizing conditions.

### Behavioral Task

#### Participants.

127 total subjects were recruited to participate in the online experiment. All subjects were compensated by research credits for their time. Because they were recruited through anonymous means, we did not collect demographic information such as age or gender; however, participation in the University of California Irvine subject pool requires that one is enrolled as an undergraduate student. Because this is a long and repetitive task and we could not monitor subjects’ attention levels or distraction, we defined exclusion criteria on the basis of type 1 performance and completion time. Subjects were excluded if their overall performance across all conditions fell below 40% correct (chance level = 33%), or if their performance in the easiest condition did not surpass their performance in the other two (much harder) conditions (see Utility-Maximizing Conditions section for descriptions of the three *utility-maximizing conditions*). These checks were to ensure that participants were paying attention to the task and performing it in good faith rather than just randomly clicking to receive their research credits. These exclusion criteria led us to discard 96 subjects, leaving 31 remaining for the main analysis. (This is apparently a very hard, boring task to do online!)

Subjects provided informed consent in the form of a study information sheet presented in their web browser before any experimental procedures commenced, clicking a button to agree to participate. Participants could stop the experiment any time. All study procedures were approved by the University of California Irvine Institutional Review Board.

#### Procedure.

The experiment was programmed using jsPsych and hosted on a webserver, accessible via any browser. Prior to the onset of experimental procedures, the browser window was triggered to become full-screen to minimize distractions.

**Figure 1.**
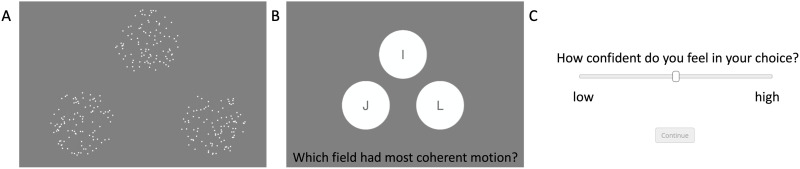
**3-alternative forced-choice (3AFC) task paradigm.** (A) Participants viewed three simultaneous random-dot kinematogram (RDK) stimuli. (B) Selected which of them contained the strongest coherent downward motion with a keypress. (C) They then used a slider to rate their confidence.

On each trial, subjects were briefly shown three random dot kinematograms (RDKs; dot-speed in visual degrees per second varied due to online presentation and varying screen sizes) of varying motion coherence in the downward direction and were asked to pick the one with highest coherence, and then report their confidence in their decision through dragging the marker on a sliding bar. Stimuli consisted of a combination of three coherences (“conditions”), one for each RDK, shown in random configuration; crucially, these stimuli were defined by the *utility-maximizing conditions* found via the model simulations (Selecting *Utility-Maximizing Conditions* and Utility-Maximizing Conditions sections). This practice gives us the best chance to identify which model may best fit the observers’ behavior, as these points were identified as the points at which type 2 behavioral predictions maximally diverged (see Selecting *Utility-Maximizing Conditions* section). Each coherence in a particular combination of coherences was equally likely to be assigned to one of the three RDKs, meaning that there was an equal probability that each RDK was the correct decision across trials: *p*(*S* = 1) = *p*(*S* = 2) = *p*(*S* = 3) = 1/3. Figure 1A shows a static example of the stimuli.

Subjects were briefly shown the three RDKs of varying coherence for 600 milliseconds, which were then immediately masked by a prompt to decide which RDK was most coherent, i.e. had the most dots moving in the downward direction (Figure 1B). Pressing [I] on the keyboard during this prompt indicated a type 1 decision in favor of the top RDK, and likewise [J] indicated the lower left RDK and [L] indicated the lower right RDK. Subjects had six seconds to respond to this prompt after the offset of the RDKs.

After making their type 1 decision, subjects were prompted to rate their confidence in their decision by sliding a marker on a bar, illustrated by Figure 1C. The left endpoint corresponded to 0 (no confidence) while the right endpoint corresponded to 100 (very high confidence). Unless subjects clicked/moved the marker on the bar, they were prevented from moving to the next trial.

The RDK, the type 1 decision, and type 2 confidence rating constituted a single trial. The experiment lasted 450 trials (150 trials per condition), and participants were given breaks every 50 trials. In its entirety, the experiment lasted approximately 51 minutes.

### Behavioral Analysis

We calculated three metrics for each observer for each condition: mean performance (% correct), mean z-scored confidence, and mean reaction time. We used z-scored confidence (z-scored within each subject across all conditions) for two reasons. First, z-scoring confidence allows us to conduct cross-participant analyses while not conflating within-subject variance and between-subject variance. That is, some subjects may have higher overall confidence than others (i.e., used different portions of the sliding-bar scale), which would inflate the between-subjects variance in confidence scores within each condition. Second, we wanted to be able to directly compare z-scored confidence between human observers’ data and model data, which had previously been z-scored for the purposes of discovering the *utility-maximizing conditions* (Selecting *Utility-Maximizing Conditions* section). (Note that in the model recovery and fitting, we check that z-scoring confidence only in the three *utility-maximizing* conditions versus across the whole simulated stimulus space does not prevent model recovery or impair fitting, which it did not; see Model Recovery section).

We tested for differences across conditions in each of these measures using one-way repeated-measures ANOVAs across condition (3 levels). Significant main effects were followed by planned pairwise contrasts as appropriate.

### Model Fitting

At each of the three conditions (points in stimulus space) tested, we compare model predictions to participants’ behavior as follows. We first must recover the amount of ‘internal noise’ present in each human observer, which controls the type 1 performance (Lu & Dosher, 1999, 2008). In the current simple models, we assume this may correspond to a single scalar multiplied by the *I*3 identity matrix Σ in Equation 4. For simplicity, we assumed zero covariance and that all variances were equal, such that fitting Σ collapses onto fitting a single scalar value *σ*_*s*_ for each subject *s* which is then multiplied by Σ such that for each subject Σ_*s*_ = *σ*_*s*_ * Σ = *σ*_*s*_ * *I*3.

We fitted *σ*_*s*_ for each subject by maximizing the goodness of fit between each model’s predicted type 1 choices and each human observer’s type 1 choices. Given that all models should produce the same type 1 decision for each simulated point, this amounts to seeking a single *σ*_*s*_ value common to all four models (we also explored potential deviations from this assumption and the effect on results; see Type 1 Behavior and Type 2 Confidence Characterization sections). We fitted *σ*_*s*_ to each subject by minimizing the cross entropy between the discrete distribution of type 1 behavioral responses produced by the model and each human observer:

$$CE_{1}=-\sum p\left(x\right)\;\log\;q\left(x\right)$$ (19)

where *q*(*x*) is the probability of producing a particular type 1 response for the model, and *p*(*x*) the same for the human observer data.

Having fit *σ* to the type 1 behavior, the critical test is to what degree each model can predict each human observer’s type 2 behavior. We thus re-ran each model using the best-fitting *σ* for each subject, and computed the cross entropy between the distribution of the model’s type 2 responses and the same responses for each human observer:

$$CE_{2}=-\sum p\left(x\right)\;\log\;q\left(x\right)$$ (20)

where *q*(*x*) is the probability of producing a particular type 2 response for the model conditioned on choice (e.g., highest coherence (correct answer), second highest coherence (incorrect answer), or lowest coherence (also incorrect)), and *p*(*x*) the same for the human observer’s data. The probability distributions of confidence conditioned on choice were approximated by Gaussian kernel density estimation to estimate the continuous empirical distributions.

To the extent that a model produces better (smaller) cross entropy once the type 2 behavior is included *using best-fitting σ*, that model can be concluded to better describe that human observer’s performance than the other models tested. We selected cross-entropy instead of log-likelihood due to the desire to fit both distributions of type 1 choices conditioned on stimulus, and continuous confidence distributions conditioned on choice and stimulus type. Cross entropy was computed using Python’s scipy package (Virtanen et al., 2020). Cross-entropy for both type 1 and type 2 responses was then compared across models using repeated measures ANOVAS across models (4 levels); significant main effects were followed by planned pairwise contrasts as appropriate. All analyses were performed via custom scripts written in Python version 3.8.2.

### Model Recovery

It is important to test whether the model fitting procedures above can correctly identify which generative model produced observed confidence behaviors when ground truth is known. We therefore performed a model recovery step to check exactly this. Each model was simulated according to the above-described simulation processes (Model Simulations section) at the three *utility-maximizing conditions* found via Selecting *Utility-Maximizing Conditions* section (see Utility-Maximizing Conditions section for these conditions’ identities), but this time with 150 trials per condition as in the behavioral experiment and *σ* chosen randomly according to a uniform distribution between 0.5 and 5 for each simulated participant. Choices and confidence judgments were recorded for each simulated participant for each trial.

We then engaged in the model-fitting procedure (Model Fitting section) and examined whether the ‘best-fitting’ model for confidence judgments (via Equation 20) was indeed the model that had generated the data. Importantly, this also involved z-scoring the confidence data *only within the three conditions chosen* to provide a direct analog to the z-scored confidence that would be computed for the human participants in these three conditions. This ensures that even though the *utility-maximizing* conditions were chosen based on z-scored confidence across *all* simulated points in the three-dimensional stimulus space, we want to ensure that the shift in range of confidence for these three points (due to other potentially very-high or very-low confidence points being excluded from the empirical experiment) does not prevent model recovery and instead serves only to move all confidence judgments into the same range across models and human participants.

## RESULTS

### Utility-Maximizing Conditions

Of all the possible combinations of three stimuli simulated, we found a number of conditions at which summed Cohen’s *d* was large (see Selecting *Utility-Maximizing Conditions* section). Because there were a number of these, we down-selected to three conditions for the sake of making the experiment practically doable (even with this, the experiment lasted ∼51 minutes online). This down-selection process also purposefully excluded conditions which led to to ambiguous type 1 correct decisions, i.e. cases where the coherences of two conditions were the same and larger than the other condition (e.g., [4.5, 5, 5]). The selected *utility-maximizing conditions* thus corresponded to:

$$\begin{array}{l} d=\left[0,0,3.5\right]\\ d=\left[3.5,4.5,5\right]\\ d=\left[4,4.5,5\right] \end{array}$$

Note that because the three-dimensional system is completely symmetric, [0, 0, 3.5] = [0, 3.5, 0] = [3.5, 0, 0] and likewise for the other two conditions. The Cohen’s *d* values for each of these stimuli, pairwise between each pair of models, are shown in Table 1.

**Table 1.** Cohen’s *d* values for the *utility-maximizing conditions* selected here.

Model 1	Model 2	[0, 0, 3.5]	[3.5, 4.5, 5]	[4, 4.5, 5]
P&L-IO	P&L-H	1.035	1.246	1.325
P&L-IO	A-IO	0.944	1.057	1.115
P&L-IO	RCE	2.089	2.835	3.182
P&L-H	A-IO	0.202	0.112	0.123
P&L-H	RCE	0.994	1.169	1.276
A-IO	RCE	1.307	1.229	1.342

Initially, we assumed that in our simulation, the point [0, 0, 5] would refer to a stimulus where one patch had 100% coherence and the other two patches had zero coherence (i.e., would correspond to a set of RDKs with coherences [0, 0, 1]). However, because during a pilot experiment (data not shown) we observed that performance was often at ceiling (i.e., 100% correct) even for *d* = [0, 0, 3.5], we scaled these *utility-maximizing conditions* by a factor of 0.16 to produce the motion coherences to be displayed in the RDKs. This produced three conditions, shown to all observers, with RDK coherences of [0, 0, 0.56], [0.56, 0.72, 0.80], and [0.64, 0.72, 0.8]. Finally, because the [0, 0, 3.5] condition would not have a meaningful ordering for which stimulus is ‘second-best’ (which is important for fitting *σ*_*s*_), in generating the physical stimuli we also slightly manipulated this condition to exhibit coherences of [0, 0.0001, .56]; this allowed us to meaningfully assign responses to ‘correct’, ‘second-best’, and ‘lowest’ for the purposes of our model fitting procedure (Model Fitting section).

### Model Recovery

Our model recovery process (Model Recovery section) was successfully able to identify which generative model had produced the confidence judgments under simulated conditions. As described above (Model Recovery section), at each of three conditions (points in stimulus space), we generated 150 trials at each *utility-maximizing condition* to generate simulated ‘participants’ as similar as possible to the empirical experiment. Given each of four models, we randomly generated stimuli by sampling from each *utility-maximizing c* (akin to the mean motion coherence of the three RDKs presented in the empirical experiment); we also randomly selected an ‘internal noise’ level for each simulated observer, that is, a *σ*_*s*_ value for each simulated observer, according to a uniform distribution between 0.5 and 5. We repeated this procedure 100 times for each model, as if we had 100 observers each with one of the four models tested as their true generating model. Finally, we tested whether our model fitting process would accurately indicate which model was the true model that had generated each simulated observer’s data.

Despite variance in fitted *σ*_*s*_ relative to the true *σ*_*s*_ due to the small number of simulated trials (purposefully matching our empirical experiment) and use of different random seeds between simulation and model fitting for each simulated subject, we observed excellent model recovery capacity from our fitting procedure. Across all simulated observers, the model recovery accuracy was 0.970 (Total: 353/364; Model 1: 91/91 = 1; Model 2: 80/91 = 0.879; Model 3: 91/91 = 1; Model 4: 91/91 = 1). Note that the total number of simulated observers is 364 and not 400 due to cases where very large (randomly-selected) *σ*_*s*_ produced infinity values in the kernel density estimates for confidence distributions; these simulated observers were thus excluded from the model recovery procedure.

### Participants’ Behavior and Model Fitting

**Figure 2.**
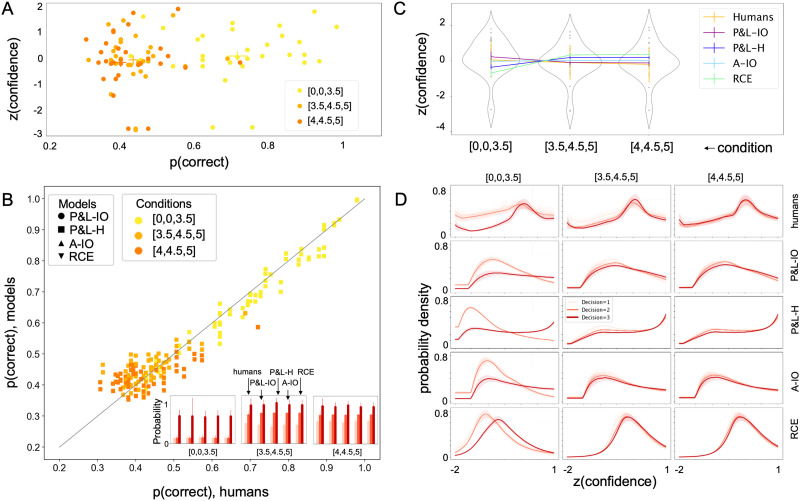
**Behavioral and model fitting results.** (A) Participants performed better in the [0, 0, 3.5] condition than in the other two conditions (also reflecting our exclusion criteria), which did not differ from each other. There was no clear relationship between confidence and accuracy. (B) Fitted models also accurately captured type 1 task performance in all conditions, shown both in the percent correct choices and in the proportion of each choice (choice = 1, choice = 2, or choice = 3 = correct). (C) Human observers’ confidence was significantly higher in the [0, 0, 3.5] condition than in the other two conditions, which were not significantly different from each other. This was relatively well captured by the models (colored lines) quantitatively—although there is a good amount of noise in the behavioral results—even though the models do make qualitatively different predictions. (D) Interestingly, though, distributions of confidence conditioned on choice differed significantly across models (as expected; bottom 4 rows), but also deviated from distributions of confidence conditioned on choice for human observers (top row).

Participants’ type 1 behavior is presented in Figure 2A. Participants performed better in the [0, 0, 3.5] condition than the other two conditions, as expected. The one-way repeated measures ANOVA on type 1 performance (% correct) revealed that participants perform significantly better in the [0, 0, 3.5] condition than in the [3.5, 4.5, 5] and [4, 4.5, 5] conditions (*F*(2, 58) = 137.284, *p* < .001; Table 2) (this is also expected given our exclusion criteria based on model simulations). The mean type 1 correctness for [0, 0, 3.5] was 0.706 (±0.141), for [3.5, 4.5, 5] was 0.432 (±0.055), and for [4, 4.5, 5] was 0.417 (±0.066). Participants’ confidence also varied with performance, albeit much more subtlely (Figure 2A; *F*(2, 60) = 8.142, *p* < 0.001; Table 2); Figures 2B–D also show comparison to model predictions for performance and confidence based on fitted *σ*_*s*_ values; see next sections for more on the fitting results. The mean z-score of confidence for [0, 0, 3.5] was 0.143 (±1.004), for [3.5, 4.5, 5] was −0.061 (±1.000), and for [4, 4.5, 5] was −0.082 (±1.031).

**Table 2.** Results of pairwise contrasts between each pair of conditions for type 1 and type 2 behavior.

Condition 1	Condition 2	Type 1 *t*(*df* = 30), *p*	Type 2 *t*(*df* = 29), *p*
[0, 0, 3.5]	[3.5, 4.5, 5]	*t* = 13.962, *p* < 0.001*	*t* = 3.04, *p* = 0.002*
[0, 0, 3.5]	[4, 4.5, 5]	*t* = 14.709, *p* < 0.001*	*t* = 3.658, *p* < 0.001*
[3.5, 4.5, 5]	[4, 4.5, 5]	*t* = 0.747, *p* = 0.458	*t* = 0.355, *p* = 0.724

* Indicates *p* < 0.05. All significant effects survive correction for multiple comparisons by any method.

#### Type 1 Behavior.

**Figure 3.**
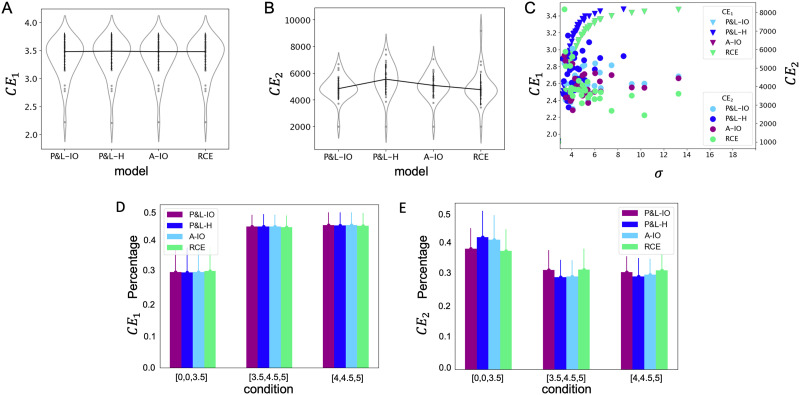
**Results of model fitting and goodness of fit.** (A) Model fitting was successful in retrieving the best-fitting *σ* for all subjects, producing (nearly) equivalent *CE*_1_ values across all four models (see main text for further clarification). (B) However, using these best-fitting *σ* values did not result in equivalent *CE*_2_ across all for models. In fact, if anything, the two “ideal observer” models (P&L-IO and A-IO) performed worse (higher *CE*_2_) than RCE, and P&L-H performed worse than all three other models. (C) This difference in *CE*_2_ is unlikely to be caused by systematic differences in *σ* across subjects, as we observed no systematic relationship between *σ* and *CE*_2_. (D) The contribution to overall *CE*_1_ was slightly lower for the [0, 0, 3.5] condition than the other two conditions. (E) The opposite was true for *CE*_2_, with [0, 0, 3.5] contributing a large amount than the other two conditions.

Maximizing model goodness of fit (minimizing cross entropy; see Model Fitting section) for type 1 behavior alone resulted in *σ* values of 3.450 on average (±2.171). With these fitted values of *σ*, all models captured type 1 behavioral performance well (Figure 2B). Cross entropy for type 1 behavior (*CE*_1_) was equivalent across three of the four models, except P&L-H (Figure 3A; repeated measures ANOVA *F*(3, 87) = 114.960, *p* < 0.001). (No outliers were present.) This is because three of the four models made the same type 1 choices under the conditions we specified (equal prior probability for each stimulus being the target, equal variance in each condition’s generating distribution, and zero covariance), but P&L-H was observed to produce small deviations in type 1 choices under the [0, 0, 3.5] condition due to the process described in Equation 9); this is because P&L-H produces slightly different type 1 choices when samples are drawn near to the origin but have negative value along one or more dimension.

We explored this main effect with planned pairwise contrasts, which revealed that P&L-H fit worse than the other models, which were equivalent to each other (Table 3). For a fair comparison based on type 2 behavior, we also checked whether allowing *σ* to vary in the P&L-H model would produce a different pattern in *CE*_1_ as when we enforce equal *σ* across all models, which it did not (repeated measures ANOVA *F*(3, 87) = 8.784, *p* < 0.001; see Table 4 for planned pairwise contrasts). (No outliers were present.) For completeness, we therefore performed the *CE*_2_ analysis with both a fixed *σ* across all four models and with allowing *σ* to vary by model.

**Table 3.** Results of pairwise contrasts (Model 1 − Model 2) between each pair of models on *CE*_1_ values, with all data (no outliers were present), with fixed *σ* across all models.

Model 1	Model 2	*CE*_1_ *t*(*df* = 87), *p*
P&L-IO	P&L-H	*t* = −15.163, *p* < 0.001*
P&L-IO	A-IO	*t* = −1.574e−13, *p* = 1.000
P&L-IO	RCE	*t* = −2.057e−14, *p* = 1.000
P&L-H	A-IO	*t* = 15.163, *p* < 0.001*
P&L-H	RCE	*t* = 15.163, *p* < 0.001*
A-IO	RCE	*t* = 1.368e−13, *p* = 1.000

* Indicates *p* < 0.05. All significant effects survive correction for multiple comparisons by any method.

**Table 4.** Results of pairwise contrasts (Model 1 − Model 2) between each pair of models on *CE*_1_ values, with all data (no outliers were present), if we allow *σ* to vary as a function of model.

Model 1	Model 2	*CE*_1_ *t*(*df* = 87), *p*
P&L-IO	P&L-H	*t* = −4.191, *p* < 0.001*
P&L-IO	A-IO	*t* = −4.155e−13, *p* = 1.000
P&L-IO	RCE	*t* = −2.708e−13, *p* = 1.000
P&L-H	A-IO	*t* = 4.191, *p* < 0.001*
P&L-H	RCE	*t* = 4.191, *p* < 0.001*
A-IO	RCE	*t* = 1.447e−13, *p* = 1.000

* Indicates *p* < 0.05. All significant effects survive correction for multiple comparisons by any method.

#### Type 2 Confidence Characterization.

With equivalent performance for model’s capacity to fit type 1 behavior (with possibly small deviations for P&L-H, which made no ultimate statistical difference in *CE*_1_ goodness of fit patterns when tested; see Type 1 Behavior section), we can now turn to the critical analysis. As a reminder, the three *utility-maximizing conditions* were selected precisely because they provided maximal effect size in the predicted differences in confidence behaviors, given a fixed type 1 performance level. For example, some models predicted high confidence for [0, 0, 3.5] but low confidence for [3.5, 4.5, 5], while others predicted lower confidence for [0, 0, 3.5] than for for [3.5, 4.5, 5] (Figure 2C). Thus, the type 2 cross entropy values (*CE*_2_) produced by each model under best-fitting *σ* for each subject will reveal which model (of these four) can best capture human type 2 behavior.

Visual inspection (Figure 3B) suggests that when predicting type 2 behavior under best-fitting *σ* values, three of four computational models demonstrated overall similar fit, although the distributions appear slightly different; P&L-H appeared to demonstrate somewhat worse fit than other three models. With all data, a one-way repeated measures ANOVA on *CE*_2_ revealed a main effect of model (*F*(3, 84) = 6.993, *p* < 0.001). If we remove outliers (±3 standard deviations from the mean), a one-way repeated measures ANOVA on *CE*_2_ confirmed this result (*F*(3, 81) = 10.054, *p* < 0.001). To explore this main effect, we conducted planned pairwise contrasts between each pair of models; this analysis revealed that this main effect was likely due to significant differences observed between any pair of model comparison which included P&L-H. For any model comparison between other three models except P&L-H, no significant differences were observed (Table 5). However, with outliers removed, we did observe a trending pairwise difference between A-IO and RCE, in a direction suggesting that RCE may in fact demonstrate slightly better fit than A-IO. As an additional check, we repeated these analyses removing the constraint that fitted *σ* be the same across all four models. This process produced equivalent results to the previous fixed-*σ* analysis (repeated measures ANOVA *F*(3, 84) = 3.883, *p* = 0.012; see Table 6 for pairwise contrasts). Together, these results suggest that P&L-H demonstrated significantly worse fit to type 2 confidence behavior, and that, if anything, RCE could fit confidence behavior better than A-IO—a finding which runs counter to the conclusions drawn by Aitchison and colleagues (2015) in their original paper but which supports the findings reported in Maniscalco et al. (2016) and Peters, Thesen, et al. (2017).

**Table 5.** Results of pairwise contrasts (Model 1 − Model 2) between each pair of models on *CE*_2_ values, with all data and with outliers (±3 standard deviations from the mean) removed, with fixed *σ* across all models.

Model 1	Model 2	*CE*_2_ *t*(*df* = 84), *p*	*CE*_2_ (outliers removed) *t*(*df* = 81), *p*
P&L-IO	P&L-H	*t* = −3.593, *p* < 0.001*	*t* = −4.460, *p* < 0.001*
P&L-IO	A-IO	*t* = −0.888, *p* = 0.377	*t* = −1.121, *p* = 0.266
P&L-IO	RCE	*t* = −0.020, *p* = 0.984	*t* = 0.538, *p* = 0.592
P&L-H	A-IO	*t* = 2.076, *p* = 0.041*	*t* = 3.340, *p* = 0.001*
P&L-H	RCE	*t* = 2.994, *p* = 0.004*	*t* = 4.998, *p* < 0.001*
A-IO	RCE	*t* = 0.867, *p* = 0.388	*t* = 1.658, *p* = 0.101

* Indicates *p* < 0.05. All significant effects survive correction for multiple comparisons by any method.

**Table 6.** Results of pairwise contrasts (Model 1 − Model 2) between each pair of models on *CE*_2_ values, with all data and with outliers (±3 standard deviations from the mean) removed, if we allow *σ* to vary as a function of model.

Model 1	Model 2	*CE*_2_ *t*(*df* = 84), *p*	*CE*_2_ (outliers removed) *t*(*df* = 81), *p*
P&L-IO	P&L-H	*t* = −2.964, *p* = 0.004*	*t* = −3.269, *p* = 0.002*
P&L-IO	A-IO	*t* = −0.917, *p* = 0.362	*t* = −1.067, *p* = 0.289
P&L-IO	RCE	*t* = −0.021, *p* = 0.983	*t* = 0.512, *p* = 0.610
P&L-H	A-IO	*t* = 3.307, *p* = 0.003*	*t* = 2.202, *p* = 0.031*
P&L-H	RCE	*t* = 3.932, *p* < 0.001*	*t* = 3.780, *p* < 0.001*
A-IO	RCE	*t* = 0.896, *p* = 0.373	*t* = 1.579, *p* = 0.118

* Indicates *p* < 0.05. All significant effects survive correction for multiple comparisons by any method.

We confirmed that this difference in cross entropy for type 2 behavior is not due to systematic differences in the ability of each model to fit type 1 behavior as a function of *σ*. That is, one might be concerned that higher *σ* values (higher noise), leading to lower type 1 performance for both model and human observer, would lead to systematic biases in the degree to which a model can fit type 1 versus type 2 performance. To confirm this was not the case, we examined cross entropy for both type 1 and type 2 behavior (for all four models) as a function of fitted *σ*. We observed a systematic relationship between fitted *σ* and type 1 cross entropy as expected (higher noise leads to poorer fits because the behavior becomes more unreliable), but no systematic change in the relationship between fitted *σ* and type 1 cross entropy as a function of model. Likewise, we observed no systematic change in the relationship between fitted *σ* and type 2 cross entropy as a function of model (Figure 3C). Thus, we can be confident that any differences in the models’ capacity to capture type 2 behavior are not due to interactions or tradeoffs between ability to capture type 1 versus type 2 behavior as a function of model which varied as a function of *σ*.

We next wanted to more comprehensively understand how well each model was fitting the data. To this end, we reexamined the models’ predicted behaviors for type 2 (Figure 2C, D) responses. Importantly, distributions of confidence conditioned on choice (Figure 2D) differed significantly not only between models, but also between models and human data. These observations suggest that despite the quantitative goodness of fit comparison suggesting that P&L-H was the ‘worst’ model and RCE potentially the best, based on *CE*_2_, *none* of the models was a particularly good fit to participants’ type 2 behavior.

Finally, we examined the degree to which each of our selected *utility-maximizing conditions* was contributing to cross-entropy at both the type 1 and type 2 levels. Here we observed that [0, 0, 3.5] was the smallest contributor to *CE*_1_, but was the primary contributor to *CE*_2_.

## DISCUSSION

### Summary of Results

Here, we introduced the first steps of a *computational-rationality* approach to arbitrating models of perceptual metacognition and beyond. A variant of optimal experimental design (Smucker et al., 2018), this approach begins with comprehensive generative model comparisons to reveal the *utility-maximizing conditions* for a given experiment: the stimuli that are most diagnostic of critical differences between models. We simulated a behavioral experiment using four literature-derived models that made identical type 1 predictions for choices in a 3-alternative forced-choice experiment, but which meaningfully differed in their predictions for confidence judgments. By selecting the *utility-maximizing conditions* and presenting them in a behavioral experiment, we revealed two findings that appear to run counter to previous reports in the literature.

First, we observed that two models explored by Aitchison and colleagues (2015) (the second also having been reported by others; Maniscalco et al., 2016; Peters, Thesen, et al., 2017) showed potentially different results than reported previously. While Aitchison and colleagues reported that their ideal observer model (here: “A-IO”) provided better explanation for confidence reports than their ‘max’ model (here: “RCE”), in that paper, both models produced similar predictions at the points tested; the superior performance of A-IO over RCE reported previously was modest at best. Here, however, using the *utility-maximizing conditions* we show that while the two models also perform approximately equivalently with all data, if anything RCE may show a trending relationship towards being the better predictor of confidence behavior.

Second, we compared two other models—here, the “P&L-IO” and “P&L-H models”—previously reported to equivalently predict confidence data in a different paradigm by Peters and Lau (2015). In that paper, maximum likelihood fitting was unable to differentiate between these two models. Here, however, the two models displayed strikingly different predictive capacity, with P&L-H significantly underperforming the P&L-IO (and, indeed, underperforming the other models tested here as well).

Together, these results reveal the importance of adopting a *computational-rationality* approach to selection of paradigms and conditions. If we want to critically arbitrate among several candidate models, we cannot rely on conditions where all models make similar predictions! This point may appear obvious, but in evaluating previous literature we see that many times, experiments are not designed specifically to arbitrate two or more models. Rather, models are fitted only after an experiment has been designed and carried out, with the result being that the conditions or paradigm used may not be ideal to specifically target differences between those models.

### Interpretation and Relation to Previous Findings

The approach taken here goes one step beyond specification of null and alternative hypotheses or seeking to reveal double dissociations, as is the standard approach in all of science. The *computational-rationality* approach involves specifying a key metric of effect size indexing the difference between model predictions, and then using formal modelling approaches (analytical or simulation) to identify the precise stimuli and/or paradigms to maximize this effect size so that maximum efficiency can be achieved in arbitrating candidate models.

In a field such as perceptual metacognition, a great many models have been put forth to describe the effect of interest—each of them enjoying empirical support in the literature. For example, in addition to the models explored here, researchers have suggested that metacognitive computations rely on: Bayesian confidence (Adler & Ma, 2018a), balance of evidence across stimulus alternatives (Vickers, 1979), reaction time (Kiani et al., 2014), complete “blindness” to perceptual noise (Peters, Fesi, et al., 2017), inaccurate representation of noise statistics (Winter & Peters, 2022), fixed confidence criteria in a signal detection framework (Li et al., 2018; Rahnev et al., 2011; Solovey et al., 2015), feed-forward processing corrupted by signal loss or log-normal noise (Maniscalco & Lau, 2016; Shekhar & Rahnev, 2021), additional information drawn directly from the sensory source (Mamassian & de Gardelle, 2022), evidence supporting the decision alone (Maniscalco et al., 2016; Peters, Thesen, et al., 2017; Zylberberg et al., 2012), stimulus or attention-driven signal variability (de Gardelle & Mamassian, 2015; Denison et al., 2018), and *many* others.

Recent efforts have started to focus on directly comparing models’ fits in the perceptual confidence field, rather than testing a single model against a null model or one other alternative model. For example, Adler and Ma (2018a, 2018b) directly compared Bayesian and non-Bayesian accounts of confidence in a perceptual decision + confidence task. Likewise, Winter and Peters (2022) compared five Bayesian-derived models of noise blindness leading to metacognitive ‘illusions’ in which confidence fails to track accuracy in the visual periphery versus central vision. Mamassian and de Gardelle (2022) directly compared models which do or do not include a ‘boost’ from information drawn directly from the sensory source, uncorrupted by type 1 processing. And there are a great many others, many of which now can depend on fitting data from the Confidence Database to aid in model comparisons (Rahnev et al., 2020). A departure from these is the experiment designed specifically to test the RCE model by Maniscalco and colleagues (2016), which revealed the RCE model to better predict behavior than a ‘differencing’ model (akin conceptually to the P&L-IO and A-IO models tested here) under conditions specifically designed to tease them apart. Yet each of these lines of research, including the study by Maniscalco and colleagues (2016), has proceeded in relative isolation (with some exceptions, of course), comparing the authors’ favorite model against few others. In honesty, the senior author of this paper is among those who have operated this way!

What should happen instead is a comprehensive exploration of the space of available models, and what kinds of processes each model can target: inputs, processes, output spaces, decision policies, and so on. To the authors’ knowledge, only two recent studies have sought to comprehensively compare the space of available models in perceptual metacognition, both using very large amounts of data and with conflicting findings. Locke and colleagues (2022) compared probability-based, evidence-strength-based, and heuristic-based confidence models, citing as their motivation the observation that these metrics “are rarely tested against one another” (p. 1). They found that while their ‘heuristic’ model was considered the overall winner at the group level, the modeling results collectively suggested a great deal of heterogeneity in how participants incorporated uncertainty into their confidence judgments, with all four model types being supported by at least one observer. In a different study, Shekhar and Rahnev (2022) also compared a number of models using data from the Confidence Database (Rahnev et al., 2020), and reported that their log-normal metacognitive noise model best fit the available data. Notably, both studies used large amounts of data, yet came to differing conclusions. What might have happened had the authors of these two studies tailored, or even selected, their experiments using *utility-maximizing conditions*? The results here—using only *three* conditions rather than the massive amount of data examined in those previous studies—suggest that the RCE model, conceptually similar to Locke and colleagues’ (2022) heuristic model and others tested by Shekhar and Rahnev (2022), was not different from the ‘Bayesian ideal observer’ models and only differed from P&L-H (although it showed a trending benefit over A-IO, in the opposite direction to that shown by Aitchison and colleagues (2015)). These findings also variously conflict with other findings in the field (e.g., Maniscalco et al., 2016, 2021; Peters, Thesen, et al., 2017). It is even the case that a deep learning model trained on naturalistic data may display some of the behaviors attributed to the RCE model in the past (Webb et al., 2023), although it is not known how such a model would behave specifically on the *utility-maximizing stimuli* used in the present study. Finally, we must also note that despite the quantitative superiority of RCE’s fit observed here, even RCE did not actually fit the type 2 data particularly well (c.f. Figure 2D). Together, these results suggest that much more work needs to be done in this area, but that more data is not necessarily the answer; the data collection process needs to be curated to be specifically suited to answer the model comparison question at hand.

This factorization of models approach—and their specific points of divergence—has recently been theoretically explored by Peters (2022), and now can be put into practice starting with the approach used here. A taxonomy of models within perceptual metacognition is needed, including how they do (and do not) relate to one another. Subsequently, through analytical derivation or simulation, *utility-maximizing conditions* in common experimental approaches can be sought so that the field can optimize its exploratory process to target efficiency rather than randomly sampling the stimulus and paradigmatic landscape. Indeed, this would be similar to the approach advocated by Palminteri et al. (2017), who implore authors to “Simulate *ex ante* the two (or more) competing computational models across a large range of parameters to ensure that the task allows discrimination between the models: the models should exhibit distinct behaviors along the cognitive dimension(s) of interest over (preferentially large) parameter ranges” (p. 430). The approach we advocate here thus expands on Palminteri and colleagues’ proposition in two ways: first, with the prescriptive advice to not only ensure that a given task allows model arbitration but to *mandate* such capacity; and second, to marry this approach with the large-scale factorial model comparisons and datasets now available especially in the field of perceptual metacognition (Rahnev et al., 2020; Shekhar & Rahnev, 2022).

### Limitations

The approach and results presented here represent a preliminary first step in how to better optimize our study of metacognitive models, and models of cognition more broadly. As such, our study has a number of limitations.

First, our results themselves should not be taken to definitively ‘prove’ that one model of metacognition is best, even among the few tested here. Our results may run counter to previous findings even among comprehensive model comparison studies (Locke et al., 2022; Shekhar & Rahnev, 2022)—but these also run counter to each other! Indeed, only one model here performed quantitatively worse than the others (P&L-H), which serves to poignantly highlight the extreme reliance of model comparisons on the conditions selected for testing and their potential for producing diagnostic results. As noted above, we also note that the actual fits between all models and type 2 human data (c.f. Figure 2D) were quite poor when visually inspected, suggesting the need for further model development or assessment. And in the same vein, we also acknowledge that our models were quite simple, not accounting for all complexities or variants tested in other papers, but recall that a comprehensive model comparison is not our intent: instead, we wish to demonstrate the utility of a *computational-rationality* approach to model comparisons, both in perceptual metacognition but also more generally.

Another reason for the somewhat equivocal findings here may also be the noisiness of the dataset—as this experiment was run online during the COVID-19 pandemic, despite access to a large subject pool, the results are unfortunately quite noisy, even after we implemented our reasonable exclusion criteria. The number of subjects excluded may also reflect the reliance on the method of constant stimuli used here, rather than titrating the coherences of the RDKs to each individual participant. Future studies can expand this approach to a broader group of subjects to make up for this noisiness, or collect cleaner data in a laboratory setting, perhaps using individually-calibrated thresholds. However, as the purpose of this study is to demonstrate a proof of concept of the *computational-rationality* approach, we leave more comprehensive exploration of these models (and others) with larger or cleaner datasets to future experiments.

Another contributing factor may have been that subjects were compensated for their time but not incentivized towards high performance or to encourage their confidence to track their accuracy. While we could have incentivized performance—which may have reduced type 1 noise in the data—there are some intriguing complexities to consider in how doing so might interact with incentives (or lack thereof) for confidence. For example, it has been shown that different incentive structures or even feedback can lead to different patterns of confidence responses even when type 1 choices remain consistent (Locke et al., 2020; Maniscalco et al., 2016). Theoretical and analytic discussion of metacognitive rating strategies has also suggested that incentive structures may interact with type 2 choices and even metacognitive inefficiency in unpredictable ways (Maniscalco et al., 2022). Thus, we chose not to incentivize behavior in this study. Future studies may wish to explore how incentives may interact with model comparisons or encourage participants to adopt one strategy for rating confidence over another.

We likewise could have selected more or different *utility-maximizing conditions*, or even other models to include in the initial simulation and condition selection process. It is true that there were several other conditions at which Cohen’s d between two or more models’ confidence predictions were quite high, and it is likely that inclusion of other models would have changed which conditions were identified as *utility-maximizing*. Indeed, the results and approach here serve primarily as a first step towards a complete pipeline for *computational-rationality* approaches. A fully computational rational approach should involve both (a) exploration of other possible metrics for quantifying the utility of a particular experiment or condition, and (b) formal analytic approaches to identifying the optimal number of conditions or (c) how conditions may be combined. That is, ideally, one would specify how each additional condition or experiment would contribute to the overall utility of a particular experimental approach, and identify the number of conditions at which utility may asymptote; one should also show that this selection is not overly dependent on the metric used to compute the utility of each condition or combination of conditions. We hope that the present approach serves as inspiration for future work in this vein. Nevertheless, that our results already revealed deviations from previously-reported literature, as described above, suggests that even under this limited exploration, our approach has strong promise over previous methods. As the purpose of this investigation was to introduce the *computational-rationality* approach to model comparisons, we suggest future studies expand our findings and the models tested to reveal which model(s) provide the best explanation of type 2 behavior across a range of paradigms, stimuli, and experiments.

## SUMMARY

Here, we have shown that a *computational-rationality* approach to model arbitration in the study of perceptual metacognition shows promise for winnowing a zoo of potential confidence models into a subset of highly promising ones with minimal data needed. In particular, we showed that principled selection of *utility-maximizing conditions* can reveal novel insights into how such model arbitration may most efficiently be conducted. By using a model-driven approach to select the conditions, experimental designs, and so on that have the best chance of arbitrating models, we take one step closer to revealing which computations precisely explain perceptual metacognition, and demonstrate the power of such an approach for other model-driven fields of study.

## ACKNOWLEDGMENTS

We thank Brian Mansicalco for helpful comments in preparing this manuscript.

## AUTHORS CONTRIBUTIONS

Yingqi Rong: Conceptualization, Formal analysis, Methodology, Software, Validation, Visualization, Writing—Original draft, Writing—Review & editing. Megan A. K. Peters: Conceptualization, Formal analysis, Funding acquisition, Methodology, Project administration, Resources, Supervision, Validation, Visualization, Writing—Original draft, Writing—Review & editing.

## FUNDING INFORMATION

This work was supported in part by the Canadian Institute for Advanced Research Azrieli Global Scholars Program (to MAKP) and by the Air Force Office of Scientific Research YoungInvestigators Program FA9550-20-1-0106 (to MAKP). Funding sources had no involvement in the design and methodology of the study.

## DATA AVAILABILITY STATEMENT

All anonymized data and code for the simulations can be found at https://github.com/CNClaboratory/3afc_comprationality.
